# Effect of Loading Rate Upon Conventional Ceramic Microindentation Hardness

**DOI:** 10.6028/jres.107.023

**Published:** 2002-06-01

**Authors:** George D. Quinn, Parimal J. Patel, Isabel Lloyd

**Affiliations:** National Institute of Standards and Technology, Gaithersburg, MD 20899-8521; Weapons and Material Directorate, Army Research Laboratory, Aberdeen, MD 21005; Department of Materials and Nuclear Engineering, University of Maryland, College Park, MD 21742

**Keywords:** aluminum oxynitride, hardness, loading rate, silicon carbide

## Abstract

The world standards for conventional ceramic hardness have varying requirements for control of loading rate during the indentation cycle. A literature review suggests that loading rate may affect measured hardness in some instances. In view of the uncertainty over this issue, new experiments over a range of indentation loading rates were performed on a steel, sintered silicon carbide, and an aluminum oxynitride. There was negligible effect upon Vickers hardness when loading rate was varied by almost four orders of magnitude from approximately 0.03 N/s to 10 N/s.

## 1. Introduction

The majority of conventional microindentation hardness data is collected under quasi-static conditions. Indenter penetration rate is set to a nominal indenter velocity or loading rate. Usually the rate is slow enough to avoid impact, yet fast enough that test cycle times are practical. Impact causes underestimates of hardness if transient load spikes create oversized indentations. Many conventional hardness machines have dashpots, solenoids, or other mechanisms to apply load smoothly and avoid impact. Researchers have either focused on minimizing rate effects as sources of experimental error in measuring static hardness, or alternatively, have studied whether there is a genuine rate effect in the material’s hardness response. A short review follows.

Tabor’s 1951 seminal work [1] included a chapter on dynamic hardness that was defined as the resistance of the metal to local indentation produced by a rapidly moving indenter. The discussion was limited to dropped-ball impact hardness testing. The dynamic contact pressure at yielding was considered a dynamic harness number. Contact pressures could be estimated by comparing the kinetic energy of the impacting indenter (less the rebound energy) to the energy to produce the indentation volume. This energy or work per unit volume is analogous to hardness; hardness has units of force per area and multiplying the numerator and denominator by the depth of penetration converts hardness to units of energy per volume. An alternative analysis using rebound height measurements could also estimate the yield pressure and dynamic hardness. Tabor recognized that the forces, displacements, and time history during impact indentation could be quite complicated and he had to begin with some simplifying assumptions. His first model assumed that dynamic yield pressure was constant throughout the process of impact. This ignored dynamic effects that increase the pressure during the initial stage of impact. Elastic waves set up in the indenter and the metal specimen were assumed to absorb negligible energy. Temperature increases in the specimen were assumed to be negligible. With these assumptions, the rebound height analysis indicated that dynamic hardness was close to static hardness values. The kinetic energy analysis produced dynamic hardness values that were higher than the static hardness. The difference, which was at least a few percent (but could be greater in softer materials) was attributed to additional forces associated with viscous flow of the metal around the indentation. Tabor added a cautionary remark about testing in general: “If therefore, in static hardness measurements, there is any jolt or impact while the load is being applied the indentation will be larger than it should be and the deduced static hardness will be lower than the true value. It is thus evident that for satisfactory static hardness measurements the load must be applied very slowly and smoothly.”

Mott [2], in his comprehensive 1956 survey of indentation testing, reviewed the effects of indenter inertia and impact errors on measured hardness. Errors due to inertia effects were shown to be proportional to the kinetic energy of the indenter mechanism normalized by the indentation load times the diagonal size. Larger errors occur for lower loads and faster indenters. McHolm [3] similarly showed that the kinetic energy of a moving indenter adds to the available work that otherwise would be imparted to the test piece by quasi-static loading. For an assumed indenter mass of 100 g and a test force of 1.96 N, kinetic energy could cause a 5 % decrease in effective Vickers hardness if indenter velocities exceed 150 μm/s. Early work suggested indenter velocities of 10 μm/s to 15 μm/s were prudent, although Knoop et al. [4] found that speeds of up to 300 μm/s were satisfactory. Knoop’s early apparatus was commercialized in 1940 but a dashpot was soon added to slow the indenter [5].

Modern standard methods for static hardness measurements limit the rate of load application, but there is considerable variability in the requirements (Table 1). The requirements are limitations to the rate of indenter motion, the loading cycle time, or a vague statement to avoid impact. Most machines in the United States are set to the American Society for Testing and Materials (ASTM) E 384 specifications. It is often overlooked that the specified indenter velocities in the glass standards are 4 to 16 times slower than the common standard rates for most other materials. Glass hardness tests use a Knoop indenter at very low loads (≈1 N) and consequently make very small, shallow impressions.

In some instances, a material’s response to penetration under fast dynamic conditions is of interest. The extent of plastic deformation and dislocation movement, densification, micro fissuring, shear faulting, twinning, or crushing may be rate sensitive. In metals, the extent of strain hardening is affected by the loading rate. The literature has mixed results on the effect of loading rate upon ceramic hardness. Some work shows negligible effect of rate upon hardness while others show dramatic hardness increases with faster loading rates. There is at least one report of decreased hardness with faster loading rates.

Early dynamic hardness testing featured a “Scleroscope,” a machine invented in 1906 by A. Shore that dropped a diamond-tipped hammer 250 mm onto a test piece [6,7]. Hardness was related to the height of the rebound of the hammer. This method, which is only used nowadays for very large specimens such as steel forgings or rolls, is considered an indentation hardness test since the hammer penetrates the specimen, albeit with both elastic and plastic deformation components. The Leeb portable hardness machine also operates on a rebound principal with a springloaded tungsten carbide ball or diamond. An electronic induction coil measures the velocity of the indenter before and after impact and hardness is computed from the ratio of the velocities [7].

Modern dynamic hardness tests include high strain rate apparatus such as split Hopkinson pressure bar devices [8,9,10], or balls or indenters fired by gas guns [11,12,13]. A dilemma in projectile indenter testing is that the load history may be very complicated. The peak load and the load-time-displacement response of the material are difficult to analyze and depend upon the indenter and specimen properties. Crucial assumptions about the specimen and indenter interaction must be invoked to solve the problems. For example, it is often assumed that deformation occurs at a constant strain rate or that the specimen offers a constant resistive pressure similar to Tabor’s assumptions [1]. A recent adaptation of the split Hopkinson bar device includes a load cell mounted directly under the test piece so that direct readings of the load cycle can be obtained without recourse to modeling analysis [8–10]. Other dynamic testing utilizes conventional hardness machines that are set to faster than normal loading conditions. Some researchers have used large capacity universal testing machines that are adapted to simulate hardness testers. This is very risky since universal testing machines are notoriously difficult to control precisely, especially if they are used in displacement control mode, and load overshoots are common. Overshoots will generate larger indentations and lead to underestimates of dynamic hardness.

Seshadri et al. [14] evaluated the effect of Vickers indenter impact velocity on the conventional hardness of sintered alpha SiC and a glass ceramic. They used a hardness testing machine described as having a deadweight system counterbalanced by a dashpot, although the machine had a rather fast standard rate of 0.3 mm/s. Their work showed a negligible effect of indenter velocity over the range of 0.1 mm/s to 1.0 mm/s for a load of 49 N. A slight trend of decreasing hardness with increasing rate may have existed, but was within the uncertainty (scatter bars) of the experiments. The yield of usable indents decreased as the impact velocity increased. Unusable indentations had excessive cracking that hampered measurements of the diagonal lengths or had indentation tips that fell on large pores. The cracking is not surprising since 49 N is a high load for such a brittle material.

Yoshioka and Yoshioka [15] constructed an apparatus for Vickers indentation of soda lime glass with indenter velocities of 0 mm/s to 2.8 mm/s and with dead weight loads of 3.5 N to 13.3 N. A camera monitored the size of the growing impression during the loading cycle. Strong nonlinear effects were attributed to a viscoelastic material response. The fastest growth in indentation size occurred on initial loading and the initial penetration rate was typically 0.2 times the collision velocity. Indentation growth decelerated until the impression asymptotically reached its final size. Dynamic hardness was slightly *reduced* at faster rates. Adiabatic heating in the immediate locale of the impression was suggested as a cause of the reduced hardness at faster rates, a finding that agrees with earlier work on glass that also showed an inverse relationship between hardness and loading rate [16,17].

Höhne and Ullner [18] measured the instrumented Vickers hardness of steel, silicon nitride, and borosilicate crown glass. In this modification of the traditional Vickers hardness test, both load and displacement were monitored during the indentation cycle. Several indices of hardness were computed on the basis of the forces and the depths of penetration (as opposed to the diagonal size). Peak load was 10 N and indenter velocities of 0.01 μm/s, 1 μm/s, and 40 μm/s were used. Hardness was essentially independent of loading rate at the two slower rates. Small hardness deviations at the fastest rate were related to metrological problems in determining the initial indenter contact point.

Evans and Wilshaw [12] reported the static Vickers hardness of ZnS at 30°C and −196°C. At 30°C, dynamic hardness increased from 2.0 GPa to 2.4 GPa as displacement rates increased from 2 μm/s to 2 μm/s. Unfortunately, the indentation load was not reported. Most ceramics exhibit a strong indentation size effect whereby hardness varies with indentation load. It was also not clear what type of testing machine was used. Although they reported these Vickers hardnesses, their study was primarily concerned with dynamic sphere impact testing at very high velocities (130 m/s to 1000 m/s). Two different models for the force-time history were derived. One model used the laws of motion to correlate the projectile momentum to target penetration. A second model analyzed the transient contact in terms of the projectile velocity and target shock wave velocities and pressures. The former model seemed to correlate to the observed sphere impact damage patterns at velocities up to 160 m/s, whereas the latter approach was better at velocities greater than 400 m/s.

Chaudhri et al. [11] used a gas gun with glass and tungsten carbide balls fired at velocities up to 250 m/s to measure the dynamic hardness of MgO, LiF, and NaCl. Strain rates were estimated to be 0.6 × 10^5^/s to 4.8 × 10^5^/s. Dynamic hardness was calculated by a rebound analysis whereby a photo electronic circuit monitored the incoming and rebound velocities of the impactor. Following a similar analysis to Tabor’s, the kinetic energy of the ball less a fraction of the rebound kinetic energy was normalized by the volume of the indentation impression. The dynamic hardness of the MgO was much greater than the quasi-static hardness that was measured using a screw driven universal testing machine at a 0.5 mm/min crosshead rate. LiF had some rate sensitivity, whereas NaCl had almost none. Dislocation motion as well as cracking contributed to the rate sensitivities [11,19].

Marshall et al. [13] impacted ZnS with a cylindrical sapphire projectile with 136° facets cut on one end, simulating a Vickers diamond pyramid. The 1 mm diameter by 1.3 mm long projectile (≈0.004 g mass) was fired by an air gun at velocities from 1 m/s to 40 m/s. Results were compared to static indentations made at 100 μm/s with a conventional indenter attached to the crosshead of a testing machine. An analytical model estimated the force and contact pressure histories and the rate of deformation of the target as the projectile decelerated. With their model, the dynamic hardness value (≈5.0 GPa) estimate was much greater than the static value of 1.9 GPa. Static indentation loads were not reported but may be deduced from the indentation sizes to have ranged from 6 N to 86 N.

Subhash and associates [8–10] recently used a modified split Hopkinson bar apparatus with a Vickers indenter at strain rates up to 2.2 × 10^3^/s. A major advantage of their experimental methodology was that a load cell was able to continuously monitor load versus time. A special momentum trap was used to eliminate multiple impacts. Dynamic hardness values for six ceramics were greater than the static hardness values. The indenter velocity was 1.5 m/s based upon readouts from a special strain gage transducer mounted on the indenter. A borosilicate glass showed the greatest relative hardness increase, 25 %, at loads up to 30 N. Alumina, two aluminum nitrides, and two zirconias had a dynamic hardness from 9 % to 19 % greater than the static hardness at loads up to 600 N.

At very slow loading rates, indentation creep becomes an issue. Some studies have evaluated the effect of dwell time upon conventional hardness at room [14,20,21,22,23,24] or elevated temperatures [25,26]. Hardness either remains constant or decreases gradually with longer dwell time. Anelastic deformation, viscous creep, slow crack growth and other time dependent phenomena accounted for indentation growth.

In view of the conflicting results reviewed above, there is a concern that the rather large range of loading rates permitted in the hardness standards (Table 1) might contribute to uncertainty to the measured values of hardness. The present study was undertaken to ascertain whether the general specifications in the world’s hardness standards methods are adequate and to determine whether a change in hardness could be detected over a range of nearly three decades of loading rate (approximately 0.01 N/s to 1 N/s). Three significantly different materials were tested at a single indentation load.

## 2. Experimental Procedure

A modern conventional hardness tester that could vary loading rates over a broad range was employed [27].^1^ Load was applied by a screw-driven crosshead that was controlled by a closed-loop feedback circuit connected to a built-in 300 N load cell. The indenter was mounted directly on the load cell. Loading rate was programmed by choosing a ramp time from a 1 s minimum to a 1000 s maximum. Displacement control was used until the specimen was contacted, then the machine shifted to load control to achieve the desired loading ramp rate. The machine cycle was programmed to slow the rate just before peak load was reached in order to avoid overshoot [28]. A constant dwell time of 10 s was used for all experiments. The timing cycle was monitored with a stopwatch. The observed dwell times at full load agreed with the programmed values, but the observed ramp times were typically only 63 % of the programmed values for all settings from 1 s to 300 s. The discrepancy may have been due to a software problem in this particular testing machine. The loading rates reported in this paper are nominal values calculated as full load divided by the experimentally observed ramp time

Indentation diagonal sizes were measured with the built-in microscope using a 50×objective lens, 10×eyepieces, and either a green or blue filter. Field and aperture diaphragms were adjusted to obtain optimum indentation tip clarity. The primary source of uncertainty in an individual hardness test is the uncertainty of the diagonal length measurement. The uncertainty contributions from the indentation load, the indenter constant, and the loading rate are much less significant by comparison. One or more indentations were measured several or more times to assess the uncertainty of an individual diagonal length reading. The standard uncertainty (±1 standard deviation) of an average diagonal length measurement for one indentation was between ±0.1 μm and ±0.5 μm depending upon the material as discussed below.

Hardness was calculated from the standard formula for force divided by contact area:

$$HV=1.8544\frac{P}{d^{2}}$$ (1)

where *HV* is Vickers hardness, *P* is the applied indenter load, and *d* is the average diagonal length for an individual indentation.

**Fig. 1:**
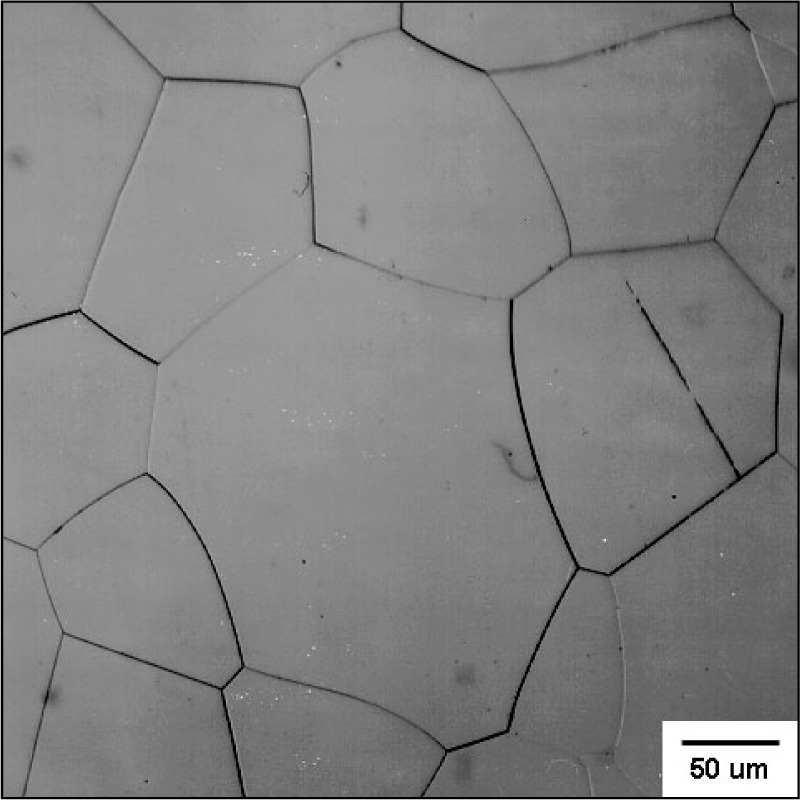
The microstructure of the coarse-grained AlON.

**Fig. 2:**
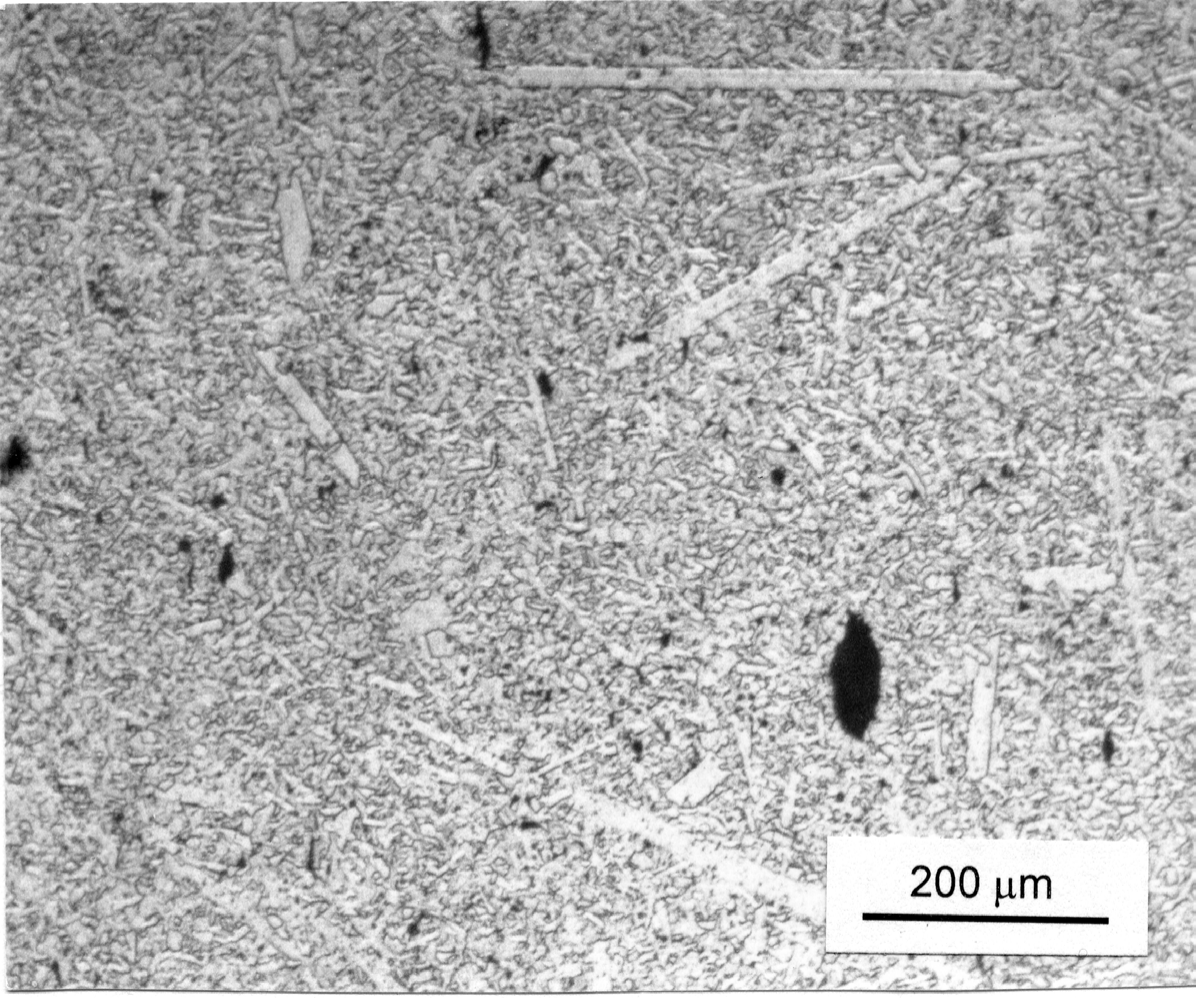
The microstructure of the sintered alpha SiC.

Three contrasting materials were tested. A steel Vickers reference hardness block was used as a baseline. Any rate effects could be due to dislocation movement or rate-sensitive strain hardening. The reference block had a certified hardness of 7.61 GPa for a 98 N load. One ceramic was a transparent AlON (Raytran grade, Raytheon Corp., Waltham, MA) that was fully dense and had nominally 200 μm equiaxed grains and no boundary phase as shown in Fig. 1. The small indentations were implanted in the middle of different grains. Individual cubic AlON grains were easy to distinguish due to relief polishing. A ceramic with much greater hardness, sintered alpha SiC (Hexaloy SA, Carborundum Co., Niagara Falls, NY) was pressure-less sintered and had a density of 3.13 g/cm^3^. The microstructure was comprised of occasional platelet alpha phase SiC grains as long as 400 μm embedded in a matrix of smaller (<10 μm) equiaxed alpha SiC grains as shown in Fig. 2. There was no boundary phase. There also was finely distributed microporosity (1 μm to 5 μm), occasional macropores (10 μm to 100 μm), and occasional spray-dry agglomerates. Indentations were randomly implanted in the specimen avoiding the large pores.

An indentation load of 4.9 N (0.5 kgf) was used for all three materials in this study. This load was used for several reasons. Firstly, severe cracking is minimized at this low load. Secondly, this load is near a critical transition point load for the two materials. Hardness of ceramics is strongly dependent upon the indentation load, an effect called the “indentation size effect (ISE)” [29,30,31,32,33]. As load is increased, ceramic hardness usually decreases until a constant hardness plateau is reached. The transition from load-dependent to loadindependent hardness may be gradual or sudden at a critical transition point [29]. Various phenomena have been proposed to account for the ISE, but increasing attention has focused on cracking in and around the indentation as a contributing factor and, in particular, a source of critical transition points. The critical transition point for a particular material is related to its indentation brittleness, *B* = *H*_c_*E*/*K*_Ic_^2^, where *H*_c_ is the critical hardness, *E* is the elastic modulus, and *K*_Ic_ is the fracture toughness [29]. The alpha SiC has a dramatic ISE transition point at 5.0 N. A fine-grained (≈25 μm) AlON studied earlier had a well-defined transition point at 7.6 N [29, 34]. The coarse-grained AlON of the present study has a critical transition point of between 1.8 N to 2.7 N. A set of experiments using the testing machine of the present study produced the latter estimate. The former transition point, were generated with a different hardness testing machine and by a different operator [30]. The two estimates are not significantly different considering the high scatter in hardness results for this material.

The polished specimen surfaces were cleaned with ethanol and dried with tissue paper prior to a test. Between 5 and 10 valid indentations (no excessive cracking and four well defined tips) were made for each condition.

## 3. Results and Discussion

**Fig. 3:**
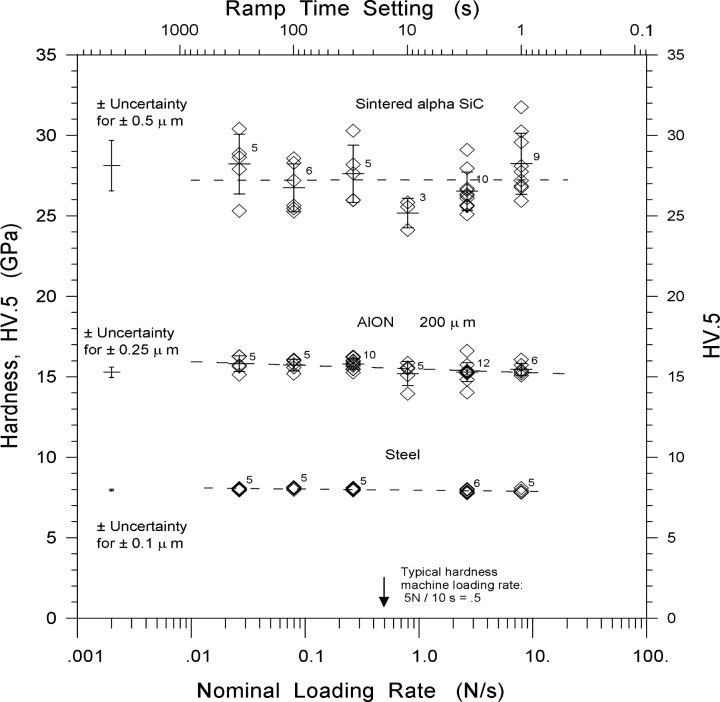
Hardness versus loading rate. The mean and standard deviation and number of indentations are shown for each test set. Much of the variability in an individual hardness result is due to the standard uncertainty (±1 standard deviation) of the average diagonal length measurement as shown on the left of each data set.

Figure 3 shows that hardness was independent of loading rate for all three materials. Any possible trend is buried within the data scatter.

The scatter in the steel hardness data is very small since the material was very uniform and indentations were easy to measure with good precision. The standard uncertainty (±1 standard deviation) of an average diagonal length measurement was 0.1 μm to 0.15 μm depending upon the test subset. The indentation size was nominally 34 μm. The low uncertainty in the diagonal length measurement yields small uncertainty in hardness as shown by the scatter bar on the left side of Fig. 3. The average hardness for 26 indentations was 7.97 GPa (±0.12 GPa, standard deviation), in good agreement with the 7.61 GPa certified value at a larger (98 N) load. The insensitivity of measured hardness to loading rate with the quasi-static rates used is not surprising for steel, but these results confirm that the new commercial testing machine functioned properly and had no overshoot at the fastest loading rates.

The greater variability in the AlON was due in part to the difficulty in measuring indentation sizes in this transparent material. There was less contrast between the indentation tips and the specimen surface. The standard uncertainty of an average diagonal length measurement was ±0.25 μm. Some of the variability was due to the different crystallographic orientations of the individual cubic grains. The 24 μm indentations were completely within individual grains in this coarsegrained AlON, and as such, the hardness values are probably single crystal values. Small cracks were at the indentation corners but did not interfere very much with the measurements. There was no obvious difference in the appearance of the indentations or their cracking propensities at the fastest and slowest rates. Nearly all the indentations were acceptable. The average hardness for all 43 indentations was 15.5 GPa (±0.52 GPa) and is slightly greater than the 14.6 GPa reported earlier for the finer-grained, not quite fully dense AlON for loads above the constant hardness plateau [34]. From the literature review in the introduction, one may conclude that oxide ceramics and glasses are likely to be vulnerable to rate effects, but the AlON of the present study had negligible loading rate sensitivity when tested under these quasi-static conditions.

The greatest scatter occurred with the silicon carbide and more indentations had to be made to obtain valid indentations. Cracking at one or more indentation corners was the most common problem, although lateral cracks spalled off the indentation sides in a few instances. Microporosity at the tips and grain dislodgements along the indentation sides also contributed to data scatter. The standard uncertainty of an average diagonal length measurement was approximately 0.5 μm. There was no obvious difference in the appearance of the indentations or their cracking patterns at the fastest and slowest rates. The typical indentation size was nominally 18 μm. The finding that Vickers hardness was rate independent concurs with the findings of Seshadri et al. [14]. Their work varied loading rate by only one decade (indenter velocities varied from 100 μm/s to 1000 μm/s) with a large indentation load of 49 N. The present work, done at 4.9 N, extends the indentation loading rate range to almost three decades. Seshadri et al. also measured static hardness at 4.9 N at a normal indenter rate. Our mean hardness of *HV* .5 of 27.2 GPa (±1.8) is in excellent agreement with their *HV* .5 value of 27.4 GPa (±3.0). The lack of rate sensitivity shown by both studies and at two different loads is not surprising in this material that is covalently bonded and has no glassy boundary phase.

The rate insensitivity shown by the quasi-static experiments reported in the present paper could be due in part to the testing machine’s programmed loading rate slowdown just prior to reaching full load. Nonetheless, the bulk of the specimen penetration and deformation occurs during the rapid portion of the loading cycle.

## 4. Conclusions

The effect of loading rate upon the conventional Vickers microindentation hardness was evaluated for three significantly different materials. *HV* .5 of a steel, an aluminum oxynitride, and a sintered silicon carbide was independent of loading rate over approximately three decades up to 7.8 N/s. Therefore, the general guidelines or specifications for loading rates in the world standard test methods for ceramic conventional hardness appear to be acceptable.

## Figures and Tables

**Table 1:** The loading rate specifications in the common world standards

Standard	Method – Material Class	Requirement
ASTM E 384	Microhardness of Materials Knoop and Vickers	Maximum indenter velocity: 0.015 mm/s to 0.070 mm/s
ASTM C 1326	Knoop Hardness of Advanced Ceramics	“
ASTM C 1327	Vickers Hardness of Advanced Ceramics	“
JIS R 1610	Vickers Hardness of Fine Ceramics	The indenter shall contact the specimen gradually so that the inertia effect is small.
ENV 843-3	Knoop and Vickers Hardness of Advanced Technical Ceramics	No limitations. Refer to ISO 9385 and ISO 146
ISO 146	Vickers Hardness, Machine Verification	The test force can be applied without shock or vibration and in such a manner that the readings are not influenced
DIN 51-225	Vickers Hardness	For loads less than 5 kg, force shall go from zero to full load in 0.3 s to 8 s
ASTM C 730	Knoop Hardness of Glass	0.0033 mm/s±0.0008 mm/s
ASTM C 849	Knoop Hardness of Whitewares	“
DIN 52 333	Knoop Hardness, Glass and Glass Ceramics	“
ISO 9385	Knoop Hardness, Glass and Glass Ceramics	“
ISO 14705	Vickers and Knoop Hardness of Fine (Advanced) Ceramics	Apply the force without shock or vibration. The approach velocity of the indenter shall not affect the hardness value. The time of initial load application for the force until the full force is reached shall not be less than 2 s nor greater than 8 s.

## Footnotes

1 Certain commercial equipment, instruments, or materials are identified in this paper to foster understanding. Such identification does not imply recommendation or endorsement by the National Institute of Standards and Technology or the Army Research Laboratory, nor does it imply that the materials or equipment identified are necessarily the best available for the purpose.
